# A Clinical Trial to Investigate the Effect of Cynatine HNS on Hair and Nail Parameters

**DOI:** 10.1155/2014/641723

**Published:** 2014-10-16

**Authors:** Christina Beer, Simon Wood, Robert H. Veghte

**Affiliations:** ^1^CB Food Consulting LLC, 320 Sherman Avenue, Salt Lake City, UT 84115, USA; ^2^Food, Nutrition and Health Program, Faculty of Land and Food Systems, University of British Columbia, 2357 Main Mall, Vancouver, BC, Canada V6T 1Z4; ^3^School of Public Health, Faculty of Health Sciences, Curtin University, Kent Street, Bentley, WA 6102, Australia; ^4^Roxlor Global LLC, 1013 Centre Road, Suite 106, Wilmington, DE 19805, USA

## Abstract

*Objective*. A new, novel product, Cynatine HNS, was evaluated for its effects as a supplement for improving various aspects of hair and nails in a randomized, double-blind, placebo-controlled clinical trial. *Methods*. A total of 50 females were included and randomized into two groups. The active group (*n* = 25) received 2 capsules containing Cynatine HNS, comprised of Cynatine brand keratin (500 mg) plus vitamins and minerals, per day, and the placebo group (*n* = 25) received 2 identical capsules of maltodextrin per day for 90 days. End points for hair loss, hair growth, hair strength, amino acid composition, and hair luster were measured. End points were also measured for nail strength and the appearance of nails. *Results*. The results show that subjects taking Cynatine HNS showed statistically significant improvements in their hair and nails when compared to placebo. *Conclusion*. Cynatine HNS is an effective supplement for improving hair and nails in 90 days or less. EudraCT number is 2014-002645-22.

## 1. Introduction

In recent years, the dietary supplement use has increased both in Europe and in the USA with many physicians recommending their use [1, 2]. A survey of health professionals conducted in 2008 found that 66% of dermatologists (*n* = 300) recommended dietary supplements to patients in relation to skin, hair, and nail health and 79% of them personally used supplements [1]. The use of bioactive ingredients at concentrated doses found in dietary supplements can efficiently modulate the physiological processes better than the single ingredients in foods since heat or mechanical treatment of food before eating can enhance or reduce its bioavailability or activity [3, 4]. The benefits of food constituents may therefore differ if the same bioactive substances are present in nutraceutical formulations. In the case of nails and hairs the classical route of treatment is the use of topical application as well as shampoos. Nowadays, another means to improve nails and hairs is through oral administration (food and dietary supplements). The advantage of the oral administration route is that blood delivers nutraceutical bioactive compounds continuously to all compartments of hairs and nails.

Different studies on dietary supplements are arising in the scientific literature confirming the efficacy of dietary supplementation on maintaining and improving skin, hairs, and nails conditions. In 2007, Jacquet et al. [3] reported the efficacy of a dietary supplement containing 100 mg Shark Cartilage, 1.6 mg vitamin B_2_, 6 mg vitamin B_5_, 2 mg vitamin B_6_, 0.150 mg vitamin B_8_, and 350 mg fish oil (omega 3 PUFA) on skin, hairs, and nails in two open clinical trials (total of 52 women). During 58 days of this trial the product caused improvement in skin hydration, decrease of wrinkle depth/volume, a significant decrease of hair loss, and an improvement of nail conditions. Authors concluded that the product was effective in improving many signs of aging, such as skin appearance, nails, and hair. Other studies demonstrate the efficacy of oral minerals (i.e., zinc and iron) [5–9], B-vitamins [7, 10, 11], and L-cystine [7, 12] on hairs and nails. Some of these studies demonstrate that oral supplementation can have a positive effect on hairs or nails while some others demonstrate that the lack of nutrient intakes with the diet has a detrimental role on hairs and nail conditions. However, some studies lack the dose-relationship effect, employed methods are not reliable or standardized, and the study design sometimes does not take the placebo group into account.

Cynatine HNS contains a protein called keratin, in a peptide form obtained by proprietary processing of New Zealand sheep wool. This novel ingredient is stable over a wide range of pH and under conditions of elevated temperature. Keratin protein is one of nature's richest sources of cysteine. Based on this, we hypothesized that Cynatine HNS may act synergistically with the cells' own antioxidant defense, boosting glutathione and other sulfur rich proteins and peptides. Keratin is the protein from which the majority of hair and nails are made.* In vitro* studies have shown that Cynatine HNS is highly bioavailable making it capable of delivering keratin peptides to the body, particularly to the hair and nails. Based on this, a randomized, double-blind, placebo-controlled study was conducted to examine the ability of Cynatine HNS to improve end points for hair loss, hair growth, hair strength, amino acid composition, and the appearance of hair on the head as well as the strength and appearance of nails.

## 2. Material and Methods

This study was a single-center, randomized, parallel group, double-blind, placebo-controlled 90-day intervention study in 50 subjects with signs of damaged hair and nails conducted at a single site in Italy (Farcoderm, University of Pavia). This clinical research study was done in accordance with the ethical principles for medical research involving human subjects (Helsinki Declaration, revised in 1983) and was approved by the internal ethical committee (Rif. 1032-11-SB). Informed consent documents were signed by all participants after study details were explained and each participant was evaluated for inclusion and exclusion criteria evaluated by dermatologists in the screening phase. Inclusion criteria for the study included being female and being between 40 and 71 years old, Caucasian, clinical signs of stressed or damaged hair, and an agreement not to use other possible cosmetic treatments which could interfere with the study. Exclusion criteria included subjects who did not fit the inclusion criteria, pregnant or breastfeeding women, use of a similar product to the active, and metabolism disorders. Once the inclusion criteria were met and consent forms were received, a screening number was assigned and entered into a screening and enrollment log. A randomization number was then given to each subject and a nonblinded employee provided the blinded examiner with the correct product at the beginning of each treatment period.

Once subjects were enrolled in the study they were provided with a base shampoo and conditioner to standardize the cosmetic habits for evaluation of product effects on hair as well as instructions. They were asked to use the base shampoo for five days and to return for their baseline visit (Day 0). At baseline, the dermatologist rechecked compliance of subjects to the protocol, evaluated baseline value for endpoints to be measured, hair (pull test, anagen/telogen evaluation, amino acid composition, mechanical properties, and appearance) and nails (clinical evaluation for nail status and breakage tendency), and supplied subjects with either active or placebo capsules and other information needed. A daily diary was also maintained in order to evaluate the habits of the volunteers in regard to foods and drinks during the first two weeks of the study as well as tobacco habits. At the end of the study period a questionnaire was filled out regarding the participants' personal opinion about the treatment (tolerability, acceptability, and efficacy). Subjects were asked to return to have the same endpoints for hair and nails measured at 30, 60, and 90 days.

The investigational product Cynatine HNS and placebo, provided by Roxlor Global, LLC, were given to the subjects as capsules packaged in blister packages. All Cynatine HNS capsules contained 250 mg Cynatine (keratin), 7.5 mg zinc, 9.0 mg vitamin B_3_, 0.825 mg copper, 6.84 mg vitamin B_5_, 1.0 mg vitamin B_6_, and 0.150 mg vitamin B_8_ (Biotin) on an active dose basis. Each placebo capsule, identical in size, shape, and color, contained the inactive ingredients maltodextrin 370 mg and magnesium stearate 5.0 mg. On Day 1, subjects were instructed to take two capsules daily in the morning after breakfast.

All subjects known to have started treatment and who returned to the clinic for at least one follow-up visit were included in the analyses. The Cynatine HNS group had one withdrawal after Day 30 giving an *N* value of 25 for Day 30 and 24 for Days 60 and 90. The placebo group had one withdrawal after Day 60 giving an *N* value of 25 for Days 30 and 60 and 24 for Day 90. Intragroup comparisons were made using Student's *t*-test and intergroup values were determined using Mann Whitney *U* Test.

The effects of both the active and placebo groups were measured on hair using five separate tests. These tests were a pull test, an anagen/telogen evaluation of the hair, the amino acid composition of the hair, the tensile strength of the hair, and the clinically evaluated appearance of the hair.

The pull test helps evaluate diffuse scalp hair loss. Gentle traction was exerted on a bunch of hairs (about 60) in three areas of the scalp (frontal, temporal, and occipital) and the number of extracted hairs was counted. The dermatologist takes a few strands between his/her thumb and forefinger and pulls them gently. In anagen phase, growing hair should remain rooted in place while hair in the telogen phase should come out easily. If the number of lost hairs is greater than 9, pull test is positive and suggestive of telogen effluvium. The subjects were asked to refrain from washing their hair 2-3 days before the pull test.

Anagen/telogen testing is performed by choosing a targeted area (mid-vertex) of approximately 1.8 cm^2^ for clipping hair, which was dyed for gray and fair colored hair. Close-up digital photographs were immediately taken after shaving and 2 days later. The two photographs were compared by software that was able to determine if hair was in anagen phase (growing) or telogen phase (not growing). For the amino acid composition of the hair, hair samples were hydrolyzed in 6 M HCl aqueous solution. Amino acids were then separated by reverse-phase liquid chromatography and identified in an X-LC fluorimeter (model 3020FP). The amino acids measured for this test are serine, glutamic acid, cystine, and methionine. The breakage force of a single hair fiber was evaluated by a dynamometer (Tensolab 2512A, Mesdan Lab). An average of 10 readings is reported.

The hair appearance is evaluated by a licensed dermatologist who assigns a value of one to three based upon the subject's hair brightness and luster. A score of 1 is dull and devoid of brightness, a score of 2 is basically dull and not so bright, and a score of 3 is shiny and bright.

The nail appearance and tendency to break were evaluated by a licensed dermatologist. The appearance of the nails is recorded in 5 either/or categories. These categories are Hard/Soft, Resistant/Fragile, Broken/Not Broken, Rough/Smooth, and Yellowish/White. The nails tendency to break is evaluated on Day 0 with a score of one to three. A score of 1 indicates that nails are flaked, are broken, or have a tendency to break, a score of 2 means nails are moderately flaked, broken, and a score of 3 indicates that neither nails are flaked, broken nor do they have a tendency to break. At Days 30, 60, and 90, the nails tendency to break is measured via a four-point scale. A score of 1 is no improvement, 2 is slight improvement, 3 is moderate improvement, and 4 is remarkable improvement. For the nail tendency to break measures, a subject with a score of 3 initially is not included in the analysis as there is no room for improvement. Eight subjects on Cynatine HNS had scores of 3, which lowers the respective *N* values by eight for each measure. Placebo had 10 subjects with initial scores of 3, lowering the respective *N* values by 10 for this calculation.

To evaluate the fact that the statistical analysis was accurate and reliable and that the sample size was large enough to detect variation of the measured parameter a* post hoc* power analysis was performed. The output of the power analysis clearly indicated that the sample sizes were large enough (power of at least 80%) to detect the differences obtained before and after treatment.

## 3. Results

### 3.1. Hair Measurement Results

**Figure 1:**
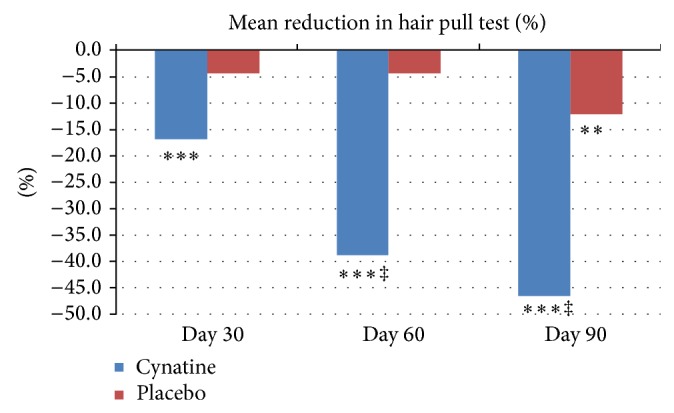
Mean percent reduction in hair pull test from baseline for placebo and Cynatine HNS. ^∗∗^ *P* < 0.01 and ^∗∗∗^ *P* < 0.001 within group to baseline; ^‡^ *P* < 0.001 between groups to baseline.

*Hair Pull Test*. The subjects in the placebo group showed no change in number of hairs lost during the study time points 30 and 60 days. However, at the end of the study period there was a significant improvement compared to baseline (*P* < 0.01). Subjects on Cynatine HNS showed a statistically significant improvement in reducing hair loss throughout the test period. A statistically significant improvement was already seen within the Cynatine HNS group at Day 30 (*P* < 0.001) with a 16.9% improvement. This further improved within the Cynatine HNS group at Day 60 (38.9%, *P* < 0.001) and Day 90 (46.6%, *P* < 0.001). The Cynatine HNS group was trending towards significance at Day 30 (*P* = 0.07) and was statistically significant at Days 60 and 90 (*P* < 0.001 for both) when compared to placebo. Overall, Cynatine HNS showed a 12.5% reduction in hair loss over placebo at Day 30 and a 34.5% and 34.4% reduction at Days 60 and 90, respectively.  Figure 1 shows the results for both groups over the 90-day time period.

*Anagen/Telogen Test*. The subjects in the placebo group showed no change in either anagen (growth phase) or telogen (nongrowth phase) phase of the hair cycle after 90 days. Subjects on Cynatine HNS showed statistically significant improvement in their anagen/telogen ratio. Both the telogen and anagen phases improved at Day 90 by 9.2% (*P* < 0.001) compared to baseline. This was also a statistically significant improvement compared to placebo at the end of the test period (*P* < 0.001).

**Figure 2:**
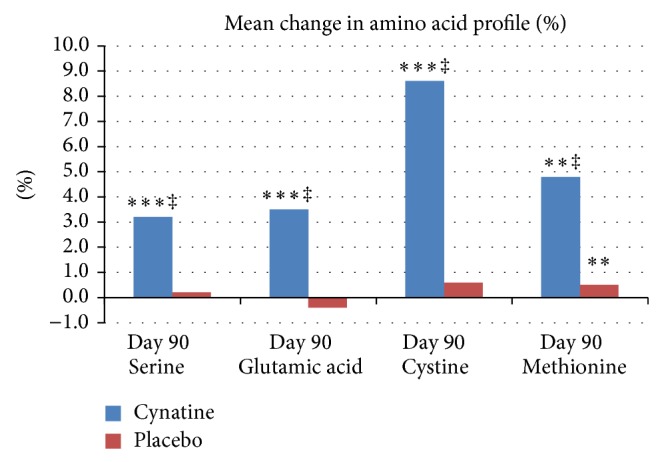
Mean percent change in amino acid profile from baseline for placebo and Cynatine HNS. ^∗∗^ *P* < 0.01 and ^∗∗∗^ *P* < 0.001 within group to baseline; ^‡^ *P* < 0.001 between groups to baseline.

*Amino Acid Profile*. Subjects taking placebo showed no improvement in the amino acid ratio of serine, glutamic acid, cystine, and methionine. At the end of the test period at 90 days the subjects on the active Cynatine HNS treatment showed a statistically significant increase in all 4 amino acids based on their ratio to total protein content. At Day 90, the mean percent increase of serine was 3.2% (*P* < 0.001), glutamic acid 3.5% (*P* < 0.001), cystine 8.6% (*P* < 0.001), and methionine 4.8% (*P* < 0.001) compared to baseline. At Day 90 these concentrations were all significantly different to placebo (*P* < 0.001). The increase of the amino acid ratio, especially of cystine which is a main component of Cynatine, also shows the bioavailability of Cynatine in the body.  Figure 2 shows the results for both groups at baseline and Day 90.

*Hair Tensile Strength*. Subjects on placebo showed no statistical improvement in their hair strength at the end of the test period. The active group treated with Cynatine HNS showed a 5.9% improvement in hair strength at Day 90 (*P* < 0.001) compared to baseline as well as a statistically significant percent change to placebo at the end of the test period (*P* < 0.001).

**Figure 3:**
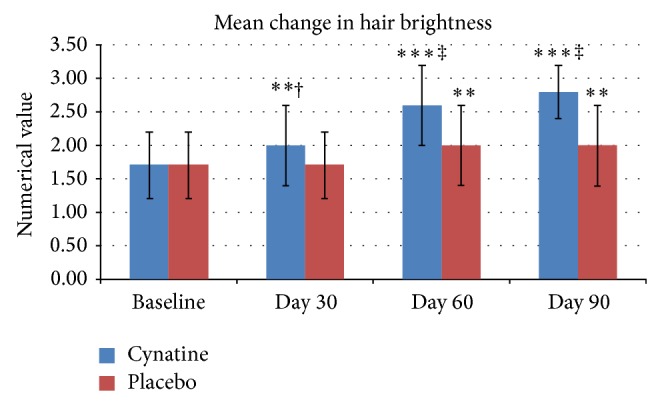
Mean change in hair brightness from baseline for placebo and Cynatine HNS. ^∗∗^ *P* < 0.01 and ^∗∗∗^ *P* < 0.001 within group to baseline; ^†^ *P* < 0.05, ^‡^ *P* < 0.001 between groups to baseline.

*Hair Appearance*. Both groups in this test started with a mean score of 1.70 ± 0.5. Subjects on placebo showed no improvement in the mean score at Day 30 and an increase of 0.30 (*P* < 0.01) at Day 60 and no further improvement at Day 90. Subjects who were treated with Cynatine HNS showed a statistically significant improvement at all times measured compared to both baseline and placebo. At Day 30 the mean increase in appearance scores was 0.30 (*P* < 0.01), at Day 60 it was 0.90 (*P* < 0.001), and at Day 90 it was 1.10 (*P* < 0.001) when compared to baseline. The results at all time points are also statistically significant to placebo (Day 30 *P* < 0.05, Day 60 *P* < 0.001, and Day 90 *P* < 0.001). The percent improvement compared to placebo was 17.6% at Day 30, 35.3% at Day 60, and 47.1% at Day 90. It also should be noted that 23 out of 24 subjects showed an improvement with Cynatine HNS, while only 8 of 24 showed any improvement on placebo. The results at all times points for both groups are shown in Figure 3.

### 3.2. Nail Measurement Results

*Nails Tendency to Break*. Subjects on placebo showed no statistical improvement over the 90-day time frame. On the improvement scale used in this test placebo had a score of 1.00 at Day 30, showing no improvement, and a mean score of 1.30 at Day 90, showing very limited improvement. Subjects on Cynatine HNS have scores of 1.90 at Day 30, 2.20 at Day 60, and 2.7 at Day 90, showing slight to moderate improvement in nails according to the grading scale used in this test. At all three time points the results compared to placebo were statistically significant (*P* < 0.001). 87.5% of subjects taking Cynatine HNS showed an improvement in their nails tendency to break, whereas only 28.5% of subjects on placebo showed any improvement.

*Appearance of Nails*. In the Hard/Soft quality of nails, hard is the desirable trait. In the placebo group, at baseline 41.7% of the subjects had hard nails and at Day 90 58.3% had hard nails. In the Cynatine HNS group, at baseline 37.5% had hard nails and at Day 90 87.5% had hard nails. A resistant nail is the beneficial quality in the Resistant/Fragile measure. At baseline, the placebo group had 41.7% of its subjects with resistant nails and at Day 90 58.3% had resistant nails. In the Cynatine HNS group, 33.3% had resistant nails at baseline and 87.5% had resistant nails at Day 90. A not broken nail is the desired result in the Broken/Not Broken measure. At baseline, 50% of the placebo group had no broken nails and 58.3% had no broken nails at Day 90. At baseline, 54.3% of the Cynatine HNS group had no broken nails and 87.5% had no broken nails at Day 90. In the Rough/Smooth measure, smooth is the desired trait. At baseline, 66.7% of the placebo group had smooth nails and at Day 90 79.2% had smooth nails. At baseline, 62.5% of the Cynatine HNS group had smooth nails and by Day 60 100% of the subjects had smooth nails. A white or natural color is desired for the nail and at baseline 83.3% had this trait in the placebo group compared to 87.5% at Day 90. 79.2% of the Cynatine HNS group had white nails at baseline and by Day 60 100% of the subjects had white nails.

All five measures of the nails appearance in the Cynatine HNS group are statistically significant to both baseline and placebo by Day 60 and all have a value of *P* < 0.02 or less at Day 90. While being still statistically significant, the *P* values in the appearance measures are larger than other measures in the study because of the limited room for improvement in many of the measures especially when compared to placebo. However, when analyzing the number of people showing improvement where possible, the largest percentage increase for placebo in any measure is 16.7%. This equates to four total people showing improvement at most in any measure on placebo. The lowest final score in the Cynatine HNS group is 87.5% in three of the measures. In those three measures, only three people total in the active group did not achieve the desired result and in the other two measures 100% of the subjects achieved the desired result.

### 3.3. Adverse Events/Withdrawals

There were no adverse events reported during the study, with 2 withdrawals. Both withdrawals were deemed by the principle examiner not to be related to either the active or the placebo group. Both withdrawals were because the subject claimed intolerance to the product, but this occurred after 30 days in the active group and 60 days in the placebo group. Based on this the examiner determined that it was individual susceptibility that was the cause of the intolerance. Both the active and the placebo groups were well tolerated in study with 100% of the subjects finishing the study saying they were well tolerated. Subjects in the active group also gave the product either an excellent or a good score in the products acceptability. Based on this, Cynatine HNS was found to be safe and well tolerated in this study.

## 4. Discussion and Conclusion

A eutrophic effect for hair on the head was seen after 3 months of treatment. This was demonstrated by the decrease of hair shedding in the pull test. The Cynatine HNS group showed significantly less hair loss after 30, 60, and 90 days which were significantly different to the placebo group. This could be explained by the improvement of the anagen and telogen phases of the hair. In the Cynatine HNS group both growth phase (anagen) and stationary phase (telogen) improved resulting in less hair being pulled out. This was not seen in the placebo group. Amino acid composition of serine, glutamic acid, cystine, and methionine improved in the Cynatine HNS group significantly to give the hair a better quality. This can be explained by the addition of the various bioavailable amino acids from keratin, which is part of the Cynatine HNS formula. With an improvement in the hair quality its mechanical properties also improved significantly at Day 90 compared to placebo. Even the clinical evaluation by the physician concluded that hair shininess and brightness had improved in the Cynatine HNS group in 87.5% of the subjects compared to only 16.7% in the placebo group. An overall assessment of hair brightness showed a 64.7% change compared to only 17.6% in placebo. This is more than a 3x improvement in hair brightness at the end of the test period.

Nails also improved their condition after 1, 2, and 3 months of treatment as demonstrated by the increase of the subjects having hard and resistant nails and the decrease of the subjects having broken and roughened nails. Hardness of nails improved from 37.5% of subjects reporting hard nails to 87.5% at the end of 90 days. That goes hand in hand with the improvement in resistance and none broken nails. The placebo group scored in all categories below 17%. As the nails improved in hardness and resistance they also improved significantly in smoothness at Day 90 compared to placebo. They also changed to a more normal color than the yellow discoloration seen. The clinical evaluation by a physician also went along the same lines and an improvement in tendency to break was seen in 87.5% of subjects as compared to only 28.6% in the placebo group.

In a questionnaire administered after completion of the study, participants were asked to rate how effective they felt the products were. Not surprisingly, the placebo scored poorly in the questionnaire as 87.5% of the participants felt that it was ineffective for hair and 84.4% felt that it was poor for nails. In the Cynatine HNS group, 91.7% of the participants felt that the product was sufficient for hair with 50% feeling that the product was either very good or excellent. For nails, 87.5% of the Cynatine HNS group felt that the product was sufficient with 66.6% finding it very good or excellent.

Based on the results of this study, it would be recommended that further clinical analysis should be performed on Cynatine HNS. In order to better analyze the effect of Cynatine HNS on hair and nails a study which includes men and women, a larger sample size, and a longer duration should be performed. Additionally it would be beneficial to look at a comparison of Cynatine with and without the additional vitamins and minerals.

In conclusion, the results obtained for the Cynatine HNS group were statistically different from that obtained for the placebo group, demonstrating that Cynatine HNS had a significant influence on the quality of skin, hair, and nails. Cynatine HNS contains ingredients that are all seen as nutrients for skin, hair, and nails. Keratin is a major structural component of the hair and nails which can be seen by the influence Cynatine HNS has on the quality of hair and nails.
